# The Progress in NHC-Catalyzed Synthesis of Organosilicon Derivatives

**DOI:** 10.3390/molecules31071108

**Published:** 2026-03-27

**Authors:** Xiaoqun Yang, Lihong Yang, Lihui Zhang, Hao Liang, Shichun Jiang, Jun Sun, Meizhong Hu

**Affiliations:** 1 School of Pharmaceutical Sciences, Tongren Polytechnic University, No. 2 Ziyou Road, Bijiang District, Tongren 554300, China; ylhhaha2025@163.com (L.Y.); lihui8852032@163.com (L.Z.); 18985337926@163.com (H.L.); 2 College of Chemistry and Chemical Engineering and Analytical and Testing Center, Guizhou University of Engineering Science, Bijie 551700, China; jiangsc@gues.edu.cn

**Keywords:** *N*-heterocyclic carbene, silylation, organosilicon derivatives, organocatalysis

## Abstract

*N*-Heterocyclic carbene (NHC) catalysis has emerged as a powerful and versatile strategy for constructing silicon derivatives, offering a metal-free alternative to traditional transition-metal methods. This review comprehensively summarizes recent advances in the NHC-catalyzed synthesis of organosilicon derivatives. Key transformations discussed include both asymmetric and non-asymmetric silylation reactions, as well as the construction of silicon-stereogenic centers. The content is systematically organized according to the types of silicon products and their underlying catalytic mechanisms. Our own perspectives on future development within this rapidly evolving field are also outlined.

## 1. Introduction

**Figure 1:**
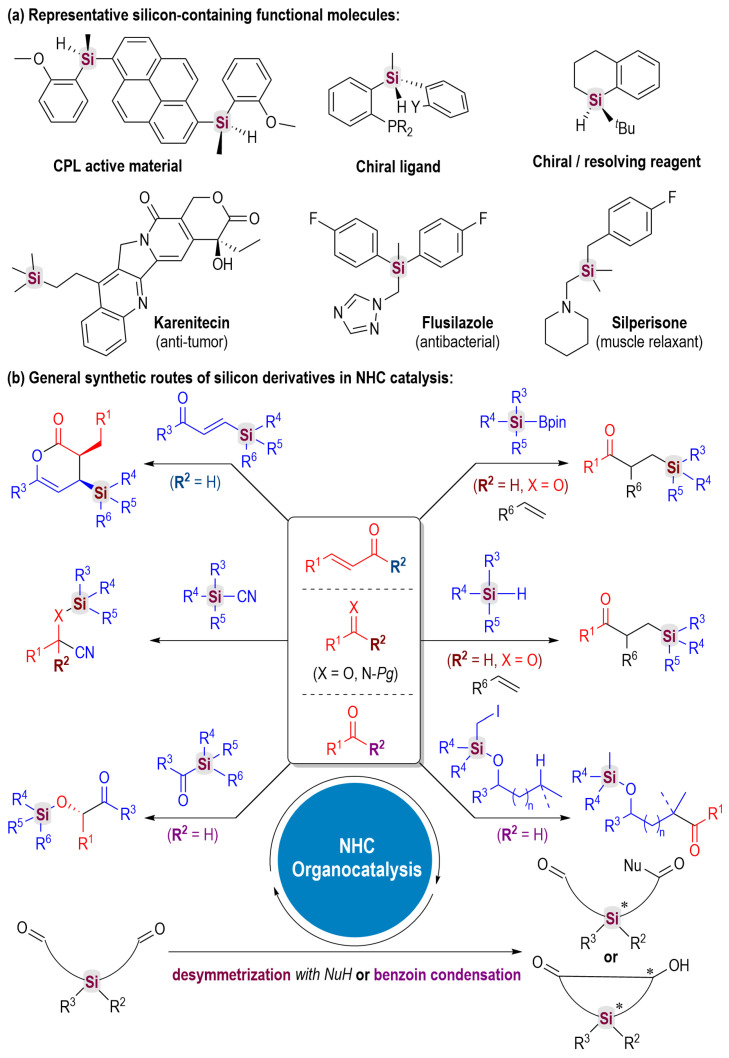
Representative silicon-containing functional molecules and NHC-mediated strategies for silicon-containing molecules; the asterisk (*) denotes chirality.

Organosilicon compounds represent a cornerstone of both modern synthetic chemistry and materials science (Figure 1a) [1,2,3,4,5]. Their unique properties, such as enhanced stability, altered electronic characteristics, and lower toxicity compared to their all-carbon analogs, underpin their widespread use from pharmaceuticals to advanced materials. Traditional methods for constructing organosilicon derivatives have heavily relied on transition-metal catalysis, particularly employing palladium, platinum, and copper complexes, which have achieved remarkable success [6,7,8,9,10,11,12]. However, these methods often entail the use of expensive and potentially toxic metal catalysts [13], stringent anhydrous and oxygen-free conditions, and challenges in controlling chemoselectivity, thereby creating an imperative for more sustainable and efficient alternatives.

In parallel, organocatalysis has emerged as a powerful and environmentally friendly tool in silicon synthetic chemistry over the past two decades [14,15,16,17,18,19]. Among the diverse organocatalytic strategies, NHC catalysis has garnered immense attention due to its ability to access unique umpolung reactivity and generate highly versatile intermediates under mild conditions [20,21,22]. While the prowess of NHCs in mediating transformations of aldehydes, esters, and other carbonyl compounds is well-established, their application in the realm of organosilicon chemistry has witnessed explosive growth [23,24]. By operating under mild, metal-free conditions, NHC catalysis enables unique transformations, including enantioselective silylation and the construction of silicon-stereogenic centers, that are difficult to achieve with traditional catalysts. This synergistic merger with organosilicon reagents provides a novel platform for the precise construction of structurally diverse silicon-containing molecules.

The catalytic cycle typically involves the nucleophilic addition of the NHC catalyst to a suitable substrate (e.g., carbonyl compounds and imines) to form a Breslow intermediate or its analogs [25,26]. This key intermediate can then engage with various silicon-based reagents through electron-pair or single electron transfer (SET) process, establishing a robust platform for diverse transformations such as C–Si bond construction, silyl ether synthesis, and silicon incorporation into complex frameworks (Figure 1b). Despite these significant advances, the field remains in its early stages, with ongoing challenges in substrate scope, catalyst design for specific transformations, and practical utility of the products.

This review aims to provide a comprehensive and timely overview of the recent progress in the NHC-catalyzed synthesis of organosilicon derivatives. The content is strategically categorized according to the types of silicon products and their associated catalytic modes, covering key transformations including silylation, sila-functionalization, and the construction of silicon-stereogenic centers. Mechanistic insights underlying these reactions and applications of the resulting silicon-containing products are also highlighted. Finally, the current challenges and future research directions in this vibrant field are outlined, with the aim of inspiring continued innovation in sustainable synthetic methodologies for organosilicon chemistry.

## 2. NHC-Catalyzed Synthesis of Racemic Organosilicons

The trialkylsilane-mediated silylation of aldehydes, ketones, and imines represents a common transformation in NHC organocatalysis, offering a powerful and direct route to value-added organosilicon compounds.

**Scheme 1:**
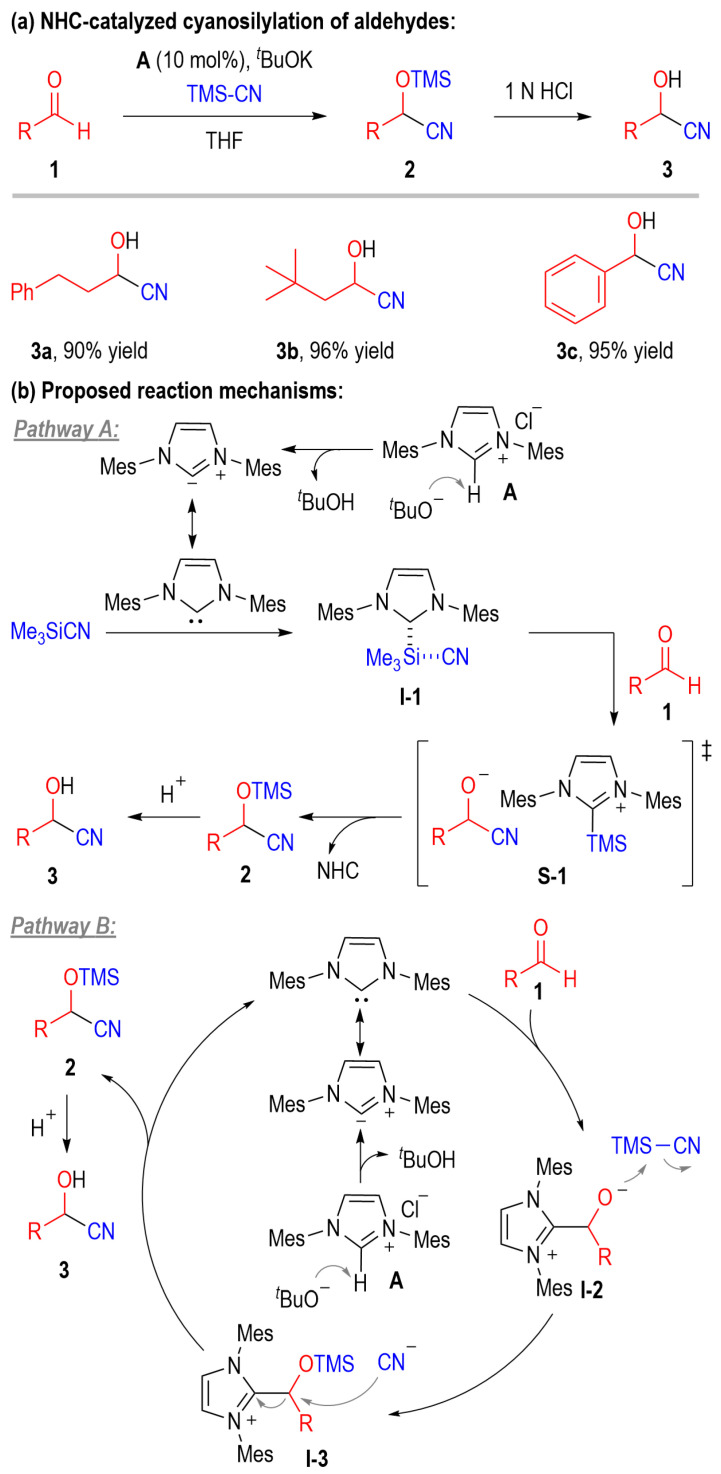
NHC-catalyzed cyanosilylation of aldehydes.

In 2006, Aoyama, Kondo, and co-workers pioneered the NHC-catalyzed cyanosilylation of aldehydes **1** with trimethylsilyl cyanide (TMSCN) (Scheme 1) [27]. This catalytic process was compatible with a wide range of aromatic and aliphatic aldehydes, affording the corresponding cyanohydrins **3a**–**3c** in 90–96% yields after acidic desilylation (Scheme 1a). Based on mechanism studies and previous reports, two plausible reaction pathways are illustrated in Scheme 1b. In pathway A, TMSCN first reacts with the free NHC **A** (10 mol%) to form the hypervalent silicate intermediate **I-1**. This intermediate then reacts with the aldehyde **1** via ion pair **S-1** to afford the cyanide **2**, with the regeneration of the NHC catalyst. Finally, a subsequent acidification yields the desired product **3**. In pathway B, the nucleophilic addition of free NHC **A** to the aldehyde **1** generates the intermediate **I-2**, which then reacts with TMSCN to give the new intermediate **I-3**. Subsequently, a nucleophilic attack of a cyanide anion to the intermediate **I-3** further deliver the compound **2** and the NHC catalyst. Acidification then affords the final product **3**.

**Scheme 2:**
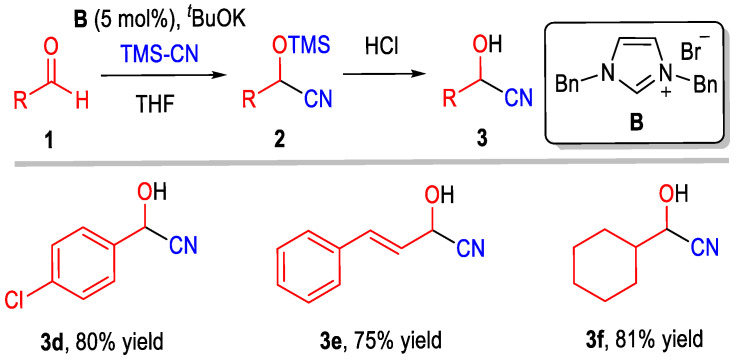
NHC-catalyzed cyanosilylation of other aldehydes.

Later, Suzuki, Sato and co-workers successfully applied the NHC catalyst **B** (5 mol%) to promote the efficient silylation of alkyl and aryl aldehydes **4** bearing substituents distinct from the previous cases, affording the corresponding cyanohydrin products **3c**–**3d** in 75–81% yields (Scheme 2) [28]. Additionally, both the pathways A and B postulated in Scheme 1b were considered plausible mechanisms contributing to this catalytic reaction.

**Scheme 3:**
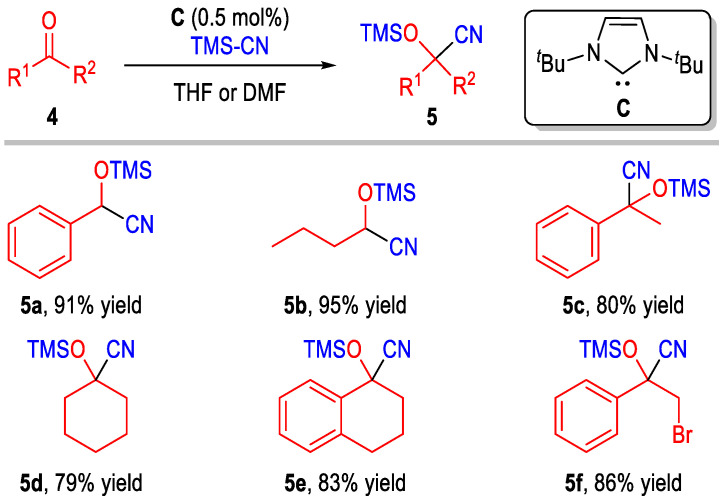
NHC-catalyzed cyanosilylation of carbonyl compounds.

In the same year, Song and co-workers discovered that beyond aldehydes, diverse ketones **4** could also serve as effective substrates within a similar catalytic strategy (Scheme 3) [29]. For example, acetophenone, cyclohexanone, α-tetralone, and 2-bromoacetophenone underwent smooth conversion under the catalysis of the free NHC **C** (0.5 mol%), with the corresponding silylated products **5** afforded in 79–95% yields. Moreover, the pathway A depicted in Scheme 1b (excluding the acidification step) was proposed as a plausible mechanism for this catalytic transformation.

**Scheme 4:**
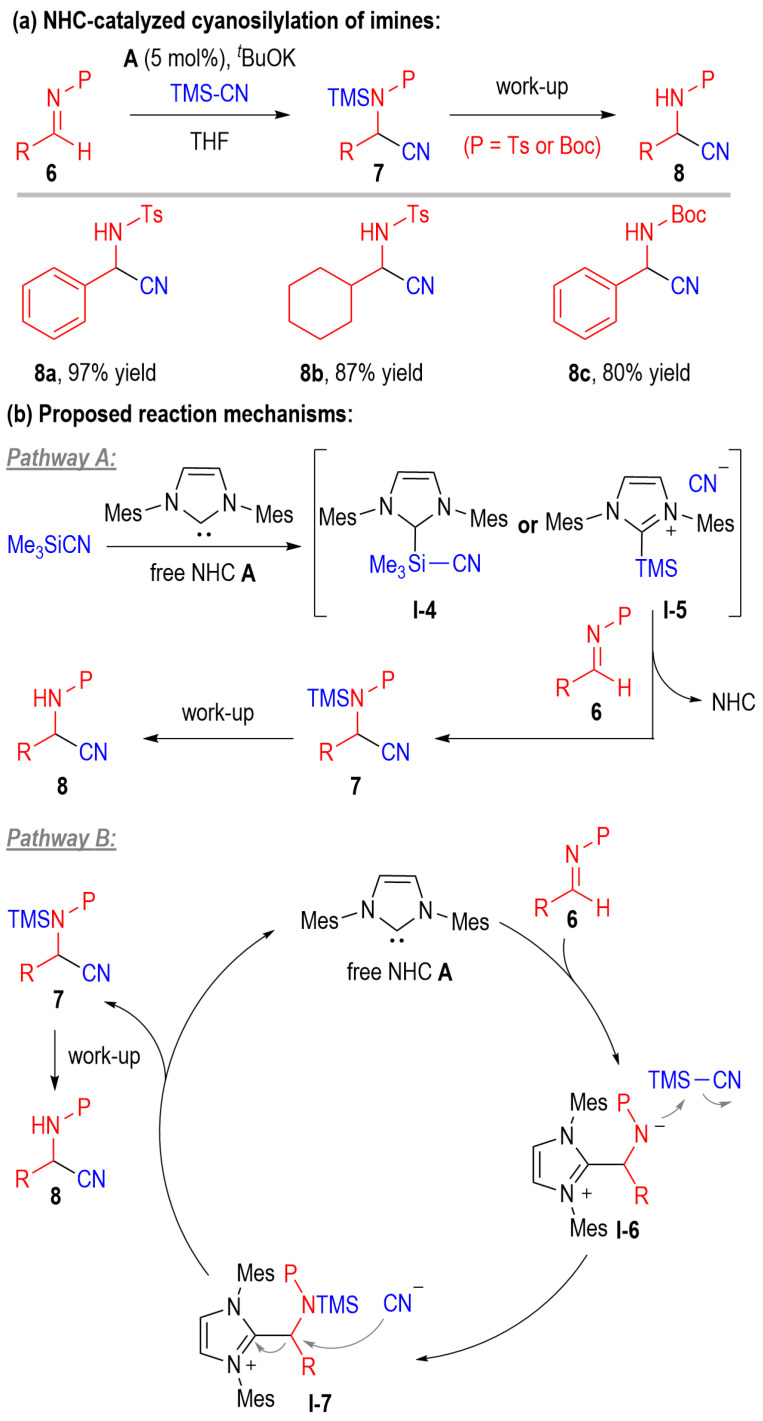
NHC-catalyzed Strecker reaction of aldimines.

Besides aldehydes and ketones, imines also serve as suitable substrates for such cyanosilylation reactions. In 2006, Aoyama and co-workers reported an NHC-catalyzed Strecker reaction of *N*-Ts or *N*-Boc protected aldimines **6** with TMSCN, affording a variety of aromatic and aliphatic α-aminonitriles **8** in 80–97% yields (Scheme 4a) [30,31]. Two plausible reaction mechanisms are postulated on the basis of experimental results (Scheme 4b). In pathway A, the free NHC **A** (5 mol%) reacts with TMSCN to form either a hypervalent silicate intermediate **I-4** or the TMS-imidazolium species **I-5**. This intermediate subsequently reacts with aldimine **6** to furnish cyanide **7** and regenerate the NHC catalyst. Finally, cyanide **7** is converted to the desired product **8**. In pathway B, the addition free NHC **A** to the aldimine **6** generates the intermediate **I-6**, which then reacts with TMSCN to give the silylated adduct **I-7**. A subsequent nucleophilic attack by a cyanide anion on **I-7** leads to the formation of compound **7** and the NHC catalyst. Upon work-up, the final product **8** is obtained. Noteworthily, this catalytic strategy was also successfully applied to ketones, yielding favorable reaction outcomes.

**Scheme 5:**
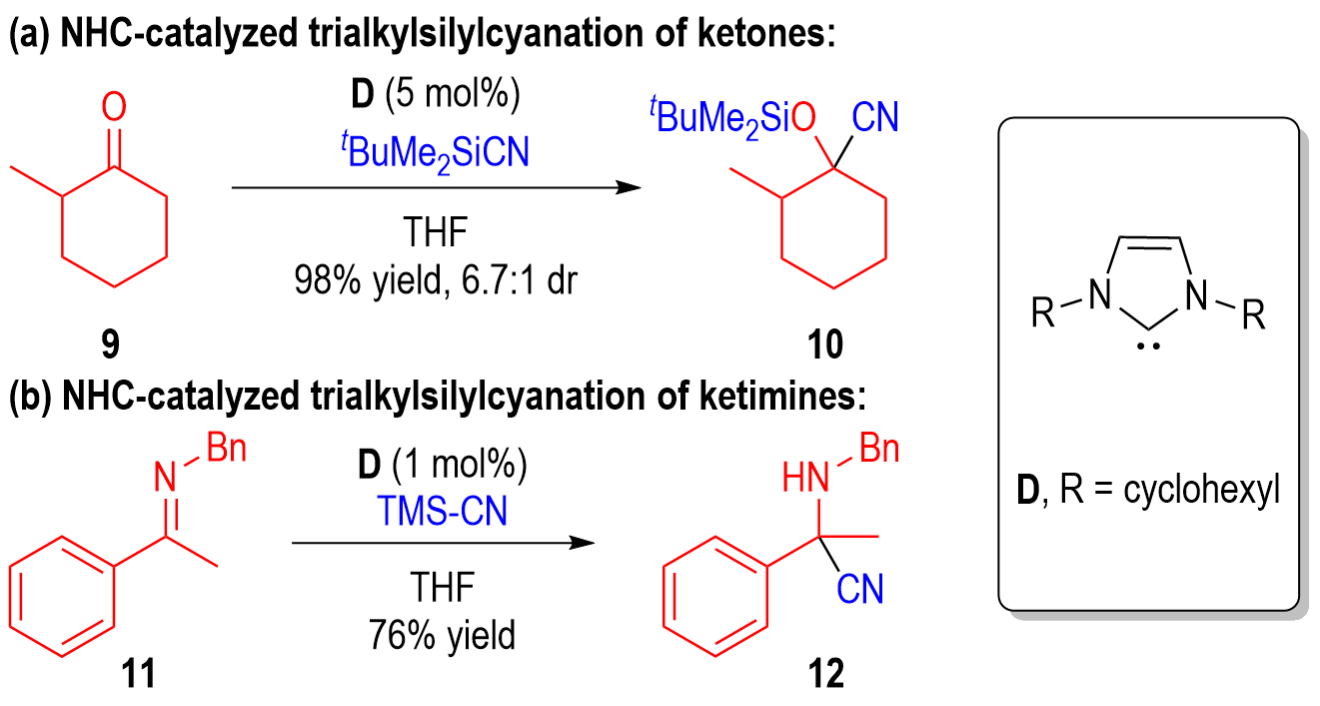
NHC-catalyzed trialkylsilylcyanation of ketones and ketimines.

Almost at the same time, Maruoka and co-workers demonstrated that a more sterically demanding trialkylsilyl cyanide could also be applied to this catalytic system [32]. Using only 5 mol% of free NHC catalyst **D**, the reaction of 2-methylcyclohexanone **9** with *^t^*BuMe_2_SiCN readily afforded the sterically congested cyanohydrin *tert*-butyldimethylsilyl ester **10** in 98% yield (Scheme 5a). Furthermore, this catalytic system also proved effective for the reaction of *N*-benzyl ketimine **11** with TMSCN, delivering the desired amino nitrile **12** in 76% yield (Scheme 5b).

**Scheme 6:**
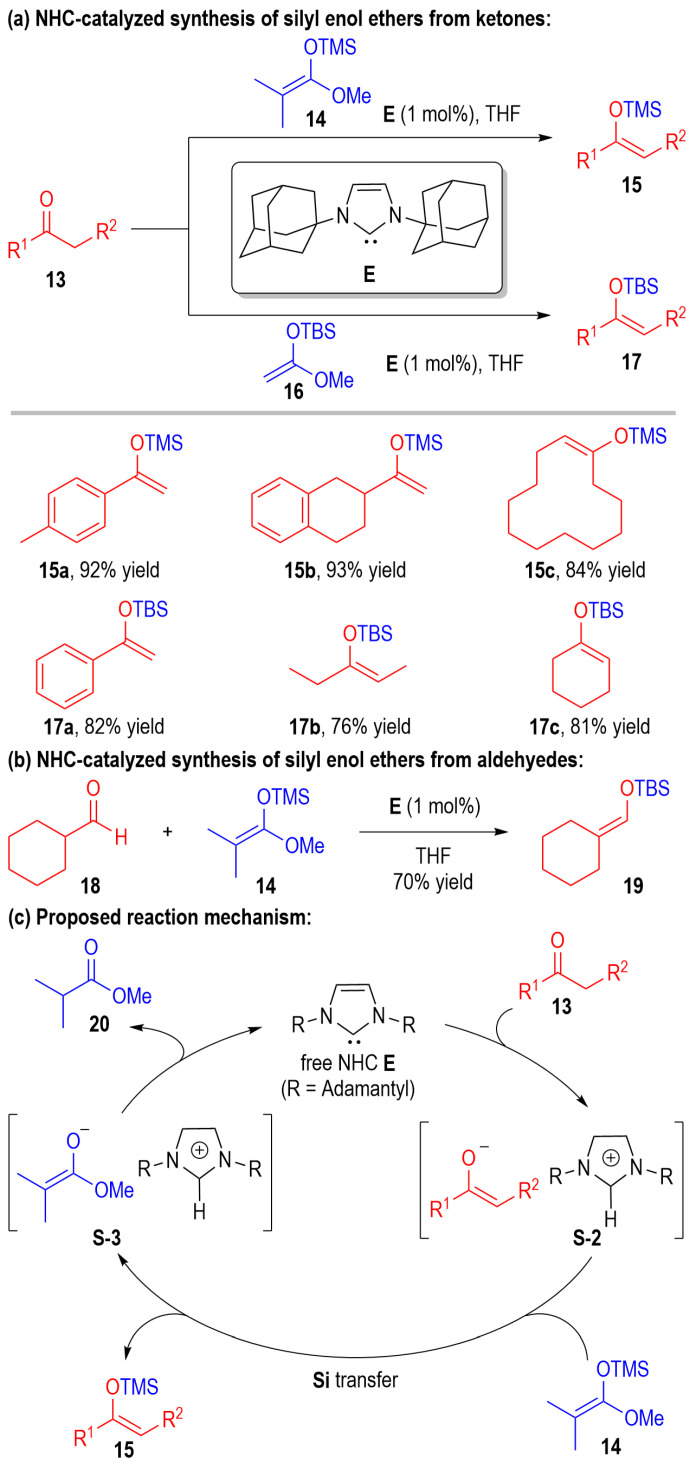
NHC-catalyzed formation of silyl enol ethers.

The NHC-promoted silyl transfer from trialkylsilyl ketene acetals **14** or **16** to ketones **15** or **17** was successfully demonstrated by Song and co-workers in 2008 [33]. Notably, this catalytic protocol accommodated ketone substrates **13** bearing diverse aryl or alkyl substitutions, delivering the corresponding products in 76–93% yields (Scheme 6a). Furthermore, the cyclohexanecarboxaldehyde **18** was also effectively converted to the corresponding TBS-protected enol ether **19** in 70% isolated yield using silyl ketene acetal **14** (Scheme 6b). Mechanistically, the deprotonation of ketone **13** by NHC **E** (1 mol%) gives the enolate species **S-2**. Subsequent silyl transfer from **14** to ion pair **S-2** occurs to form the ester enolate/imidazolium ion pair **S-3**. The final deprotonation of this imidazolium by the ester enolate regenerates the free carbene and completes the catalytic cycle, with the formation of the by-product **20** (Scheme 6c).

**Scheme 7:**
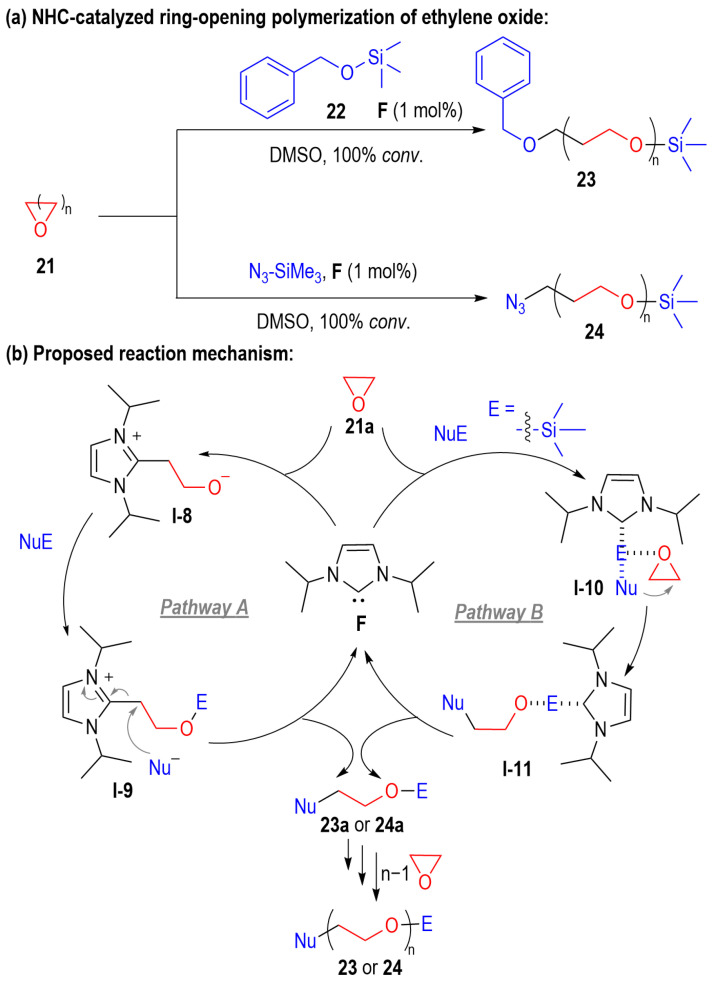
NHC-catalyzed synthesis of organosilicon polymers.

In 2010, Taton, Gnanou and co-workers reported a novel route to silylated α,ω-heterodifunctionalized poly (ethylene oxide)s **23** or **24** from ethylene oxide **21**, achieving quantitative conversion using benzyl trimethylsilyl ether **22** or trimethylsilyl azide as the silylating agents (Scheme 7a) [34]. From a mechanistic perspective, the initiation and chain growth may proceed via two distinct pathways, depending on the competition between the interaction of hydroxyl- or trimethylsilyl-containing chain regulators with the NHC catalyst **F** (1 mol%) and that of the latter with the monomer. In pathway A, nucleophilic addition of free NHC **F** to ethylene oxide **21a** generates intermediate **I-8**, which then reacts with the silylating agent (abbreviated as NuE) to afford the new intermediate **I-7**. Finally, attack by a nucleophilic anion on intermediate **I-9** releases the free NHC catalyst and affords the mono-adduct **23a** or **24a**. This mono-adduct (Nu-CH_2_CH_2_O–E) can then participate in subsequent chain propagation, a pathway referred to as the activated monomer mechanism. In pathway B, the reaction of free NHC **F** with ethylene oxide **21a** and NuE affords intermediate **I-10**, followed by an intramolecular ring-opening process to furnish intermediate **I-11**. Release of free NHC **F** from **I-11** then gives the monoadduct **23a** or **24a**, thereby completing the catalytic cycle (Scheme 7b).

**Scheme 8:**
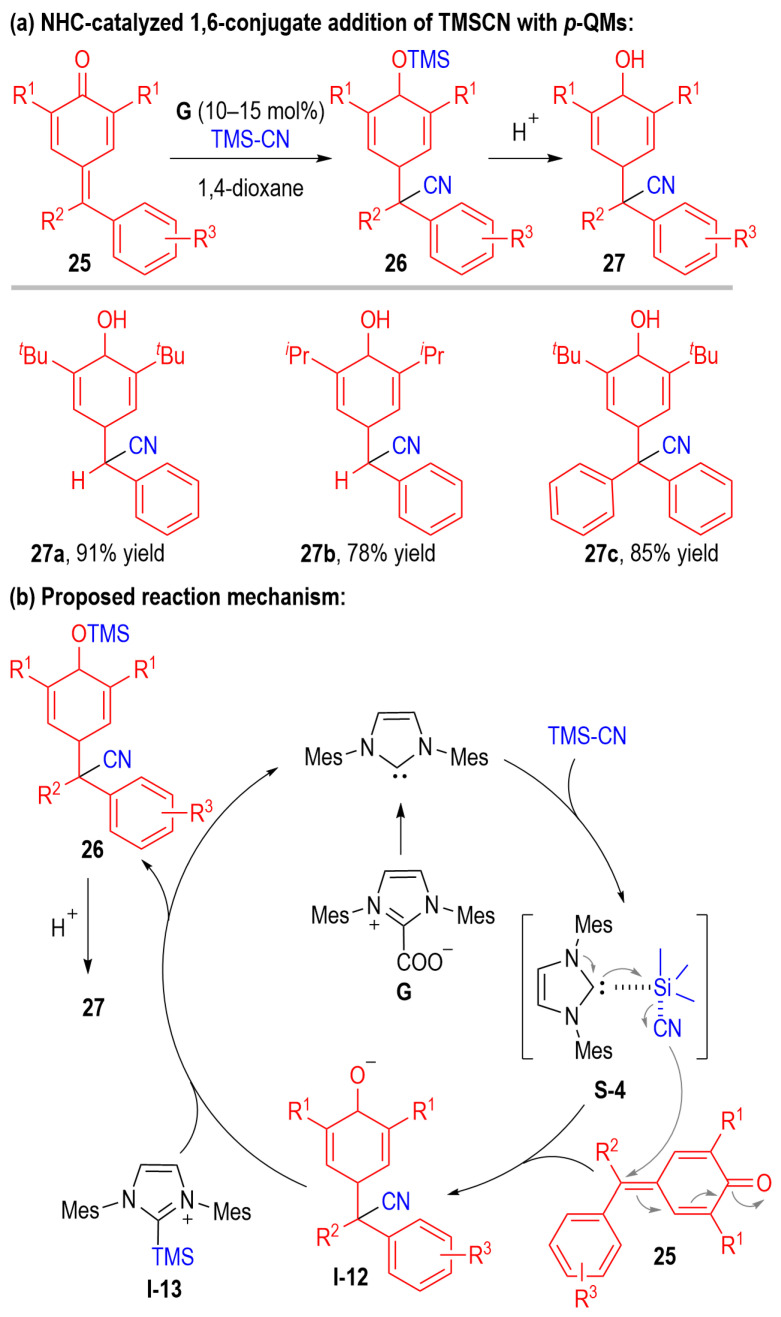
NHC-catalyzed 1,6-conjugate addition of TMSCN to *p*-QMs.

*para*-Quinone methides (*p*-QMs) serve as useful building blocks for synthesizing functionalized organosilicon molecules. In 2017, Anand and co-workers reported an NHC-catalyzed approach to α-arylated nitriles **27** (Scheme 8a) [35]. This transformation employed TMSCN as the silylating agent, affording a wide range of substituted silylated products **27** in 78–91% yields. Based on previous studies [36,37], a plausible reaction mechanism is outlined in Scheme 8b. The cycle begins with the reaction of the free NHC **G** (10–15 mol%) and TMSCN to form the species **S-4**. This addition intermediate then undergoes a nucleophilic attack on substrate **25**, yielding the intermediate **I-12**. Subsequently, a TMS group transfers from cation **I-13** to **I-12**, affording the silylated product **26** and regenerating the free NHC catalyst. Finally, acidification of **26** delivers the α-arylated nitrile **27**.

**Scheme 9:**
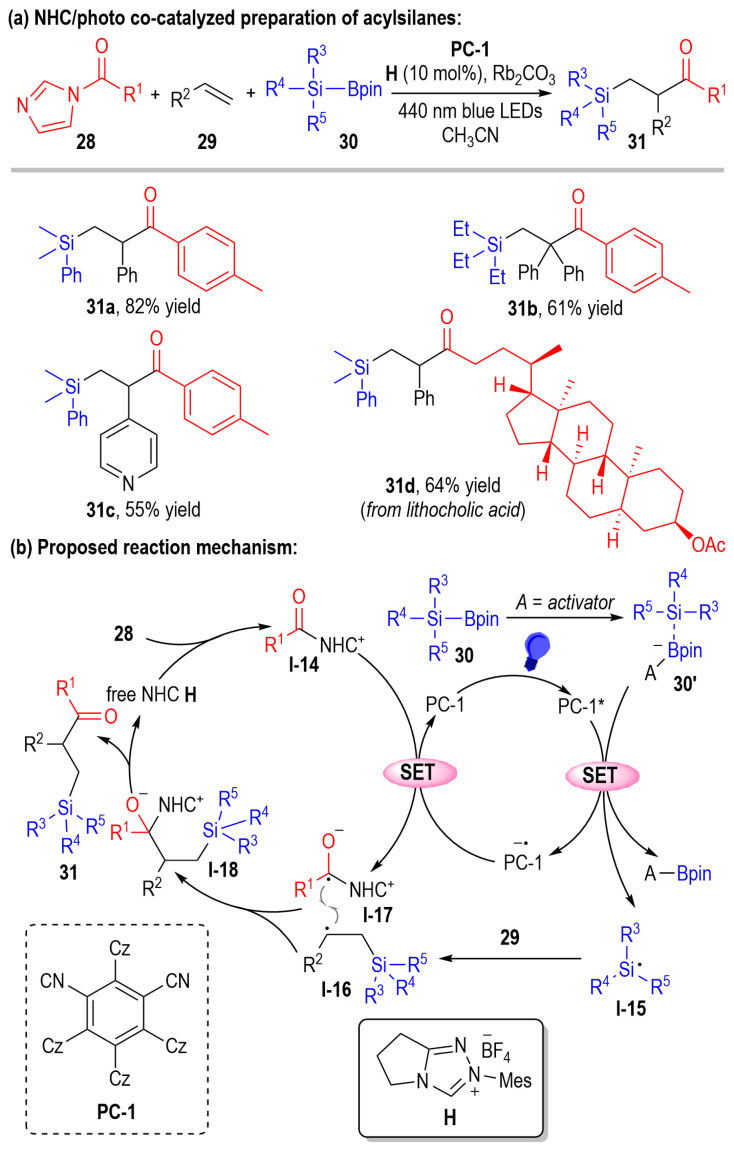
NHC/photo co-catalyzed synthesis of acylsilanes; the asterisk (*) denotes excited-state.

The NHC-involved cooperative catalysis strategy is also highly efficient for the synthesis of related functionalized silicon-containing molecules. In 2022, Ohmiya and co-workers established a visible-light-driven silyl radical generation method and applied this process with an NHC-catalyzed radical relay acylation, achieving the acylsilylation of alkenes (Scheme 9a) [38]. The reaction employed acyl imidazoles **28** as the acyl source and silylboronates **30** as the silyl source, enabling the preparation of a wide range of β-silyl ketones **31** in 55–82% yields. Notably, acyl imidazoles derived from drug molecules were also compatible substrates (e.g., **31d**).

Mechanistic investigations revealed that coordination of a DMAP molecule or a carbonate ion to the boron center of the silylboronate facilitates its oxidation. Based on this finding, a plausible mechanism was proposed (Scheme 9b). Upon oxidation by the excited-state photocatalyst, the borate adduct **30′** derived from silylboronate **30** undergoes a single-electron transfer (SET) process to liberate the silyl radical **I-15**. This radical adds across the alkene **29**, generating a carbon-centered radical **I-16**. Meanwhile, the reduced photocatalyst reduces the acylazolium intermediate **I-14** derived from the free NHC **H** (10 mol%) and acyl imidazoles **28**, furnishing the key ketyl radical **I-17** along with regenerating the ground-state photocatalyst. The subsequent radical–radical cross-coupling between **I-17** and **I-18**, followed by the elimination of the NHC catalyst, ultimately delivers the final β-silyl ketone product **31**.

**Scheme 10:**
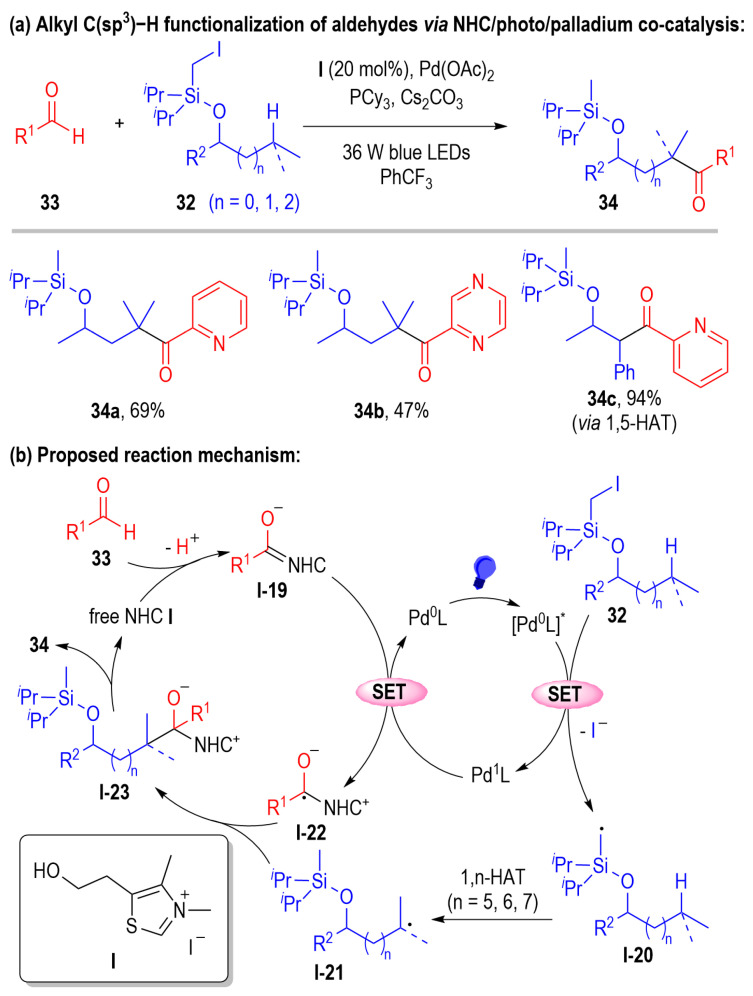
The synthesis of acylsilanes via cooperative catalytic strategy; the asterisk (*) denotes excited-state.

In addition to the use of light, the incorporation of a transition metal also serves as an effective cooperative catalytic strategy with NHCs for efficiently constructing silicon-functionalized molecules. In 2023, Ye, Zhang and co-workers introduced a cooperative catalytic strategy using photoredox, NHC **I** (20 mol%), and palladium catalysis. This approach enabled the selective synthesis of various silicon-containing ketones **34** via remote alkyl C(sp^3^)–H functionalization, delivering the products in 47–94% yield (Scheme 10a) [39]. Mechanistically, the Pd^0^L complex is excited by blue light to form the active species [Pd^0^L]*. This excited catalyst then undergoes a SET process with iodomethylsilyl ether **32** to generate a Pd^1^L intermediate and a methyl radical **I-20**, concomitant with the loss of an iodide anion. The methyl radical **I-20** subsequently undergoes an intramolecular 1,n-HAT (hydrogen atom transfer, *n* = 5, 6, 7) to form a more stable secondary or tertiary alkyl radical **I-21**. Concurrently, the Breslow intermediate anion **I-16** derived from the NHC-catalyzed activation of aldehyde **33** is oxidized by the Pd^1^L species in another SET event. This step regenerates the Pd^0^L catalyst and produces the persistent ketyl radical **I-22**. The cross-coupling between this persistent ketyl radical **I-22** and the transient alkyl radical **I-21** affords the adduct **I-23**. Finally, the adduct **I-23** fragments to release the C (sp^3^)–H acylation product **34** and regenerates the NHC catalyst (Scheme 10b).

**Scheme 11:**
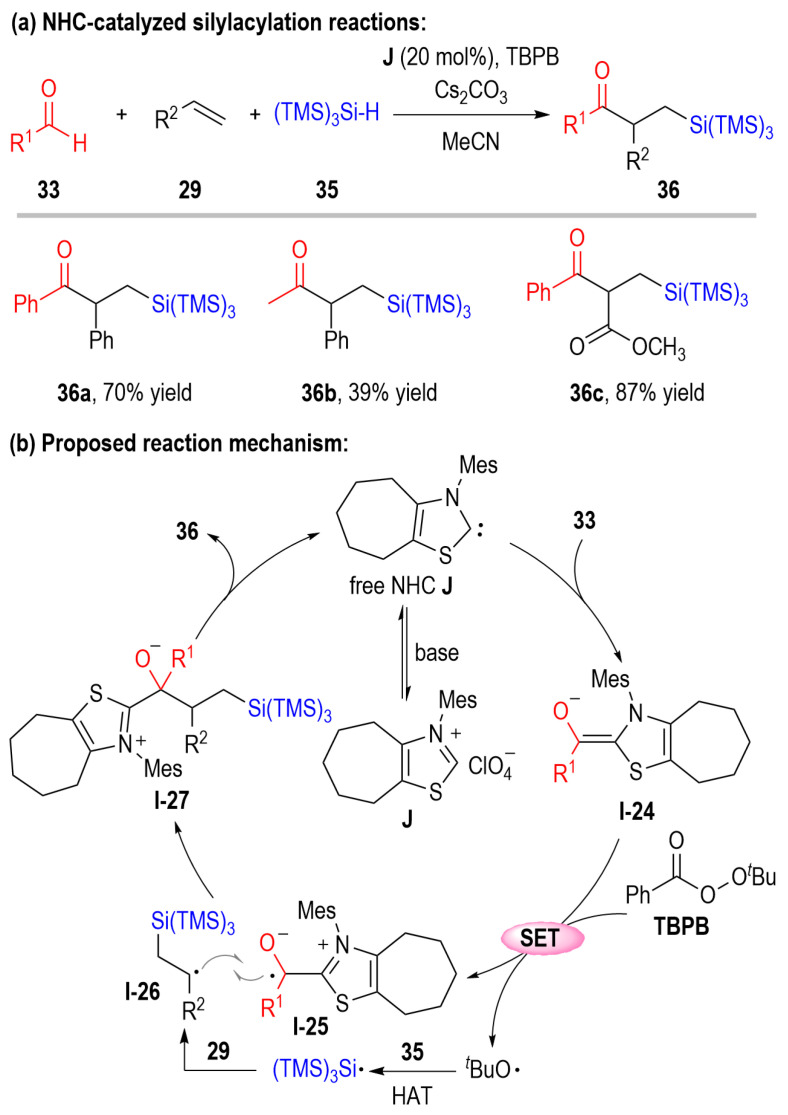
NHC-catalyzed silylacylation of alkenes.

Alkenes serve as versatile building blocks for constructing silicon-functionalized molecules through NHC organocatalysis. In 2023, Zhao, Zhou and co-workers reported a mild NHC-catalyzed method for generating silyl radicals. This approach enabled the efficient and versatile synthesis of β-silyl ketones (39–87% yield) from aldehydes **33**, alkenes **29**, and hydrosilane **35**, showcasing broad substrate scope and excellent functional group tolerance (Scheme 11a) [40]. Based on the radical clock experiments and electrochemical studies, a plausible reaction mechanism was proposed (Scheme 11b). The catalytic cycle begins with the nucleophilic addition of the free NHC catalyst **J** (20 mol%) to aldehyde **33**, forming the Breslow enolate intermediate **I-24**. This enolate then undergoes a SET reduction in TBPB, simultaneously generating the *tert*-butoxyl radical and the NHC-bound ketyl radical **I-25**. Subsequent HAT from TMS_3_SiH to the *tert*-butoxyl radical produces a silyl radical, which adds across the double bond of alkene **29**. The resulting chain-carrying benzyl radical **I-26** couples with ketyl radical **I-25** to form adduct **I-27**. Finally, release of the NHC catalyst from **I-27** furnishes the β-silyl ketone product **36** and closes the catalytic cycle.

## 3. NHC-Catalyzed Synthesis of Chiral Organosilicons

### 3.1. NHC-Catalyzed Synthesis of Carbon-Stereogenic Organosilicons

NHC catalysis can not only be used for the rapid construction of achiral silicon-containing functional molecules, but also for the catalytic synthesis of chiral silicon derivatives.

**Scheme 12:**
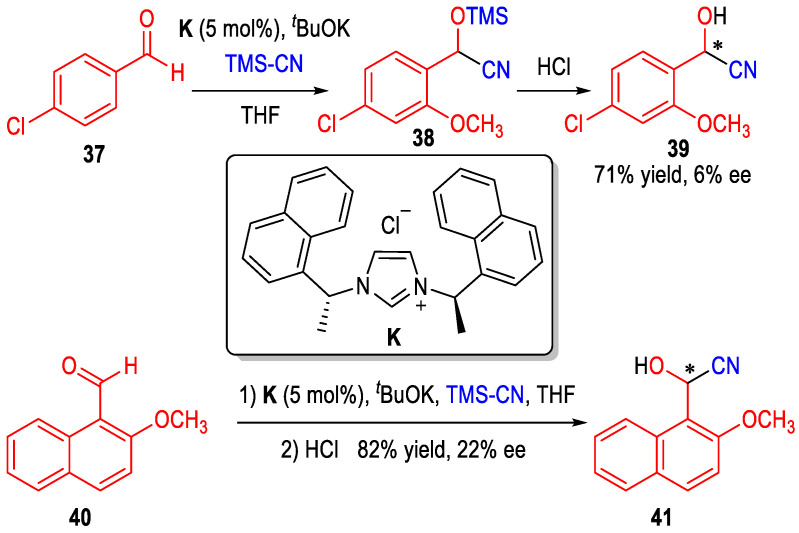
NHC-catalyzed asymmetric silylation of aldehydes; the asterisk (*) denotes chirality.

In 2006, Suzuki, Sato and co-workers proved the chiral imidazolium-based NHC catalyst **K** (5 mol%) was effective for the asymmetric silylations of aldehydes **37** and **40**, delivering the corresponding products **39** and **41** in 71% and 82% yields, albeit with poor enantioselectivity (Scheme 12) [28].

**Scheme 13:**
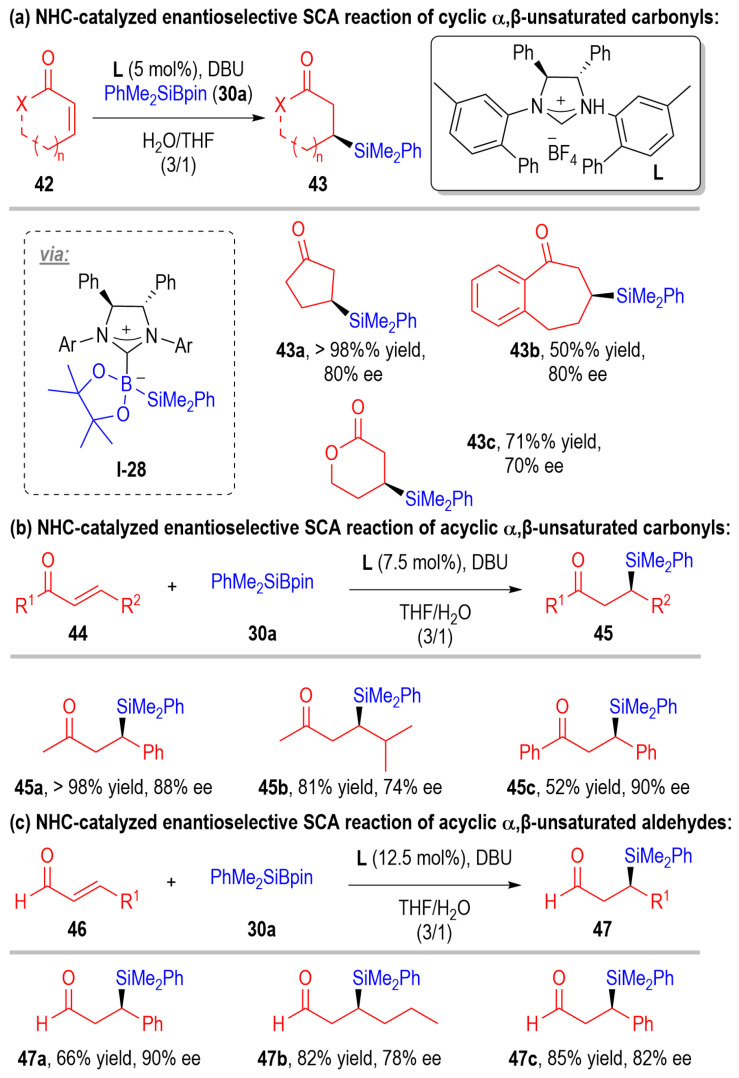
NHC-catalyzed silyl conjugate additions to cyclic and acyclic α,β-unsaturated carbonyls.

In 2011, Hoveyda and co-workers reported a metal-free, enantioselective silyl conjugate addition (SCA) reaction using 5–12.5 mol% of the designed chiral NHC catalyst **L** (Scheme 13) [41]. This methodology utilized silylboronate **30a** as the silicon source and exhibited a broad range of substrate scope, accommodating cyclic α,β-unsaturated ketones **42**, acyclic α,β-unsaturated carbonyls **44**, and α,β-unsaturated aldehydes **46**. The corresponding silylated adducts bearing either aromatic or aliphatic substituents were obtained in generally moderate to excellent yields with moderate to excellent enantioselectivities. Notably, this work established NHC catalysis as a powerful and versatile platform for enantioselective C–Si bond construction by demonstrating that intermediate **I-28**, derived from the NHC catalyst and silylboronate **30a**, serves as a key nucleophilic species. This intermediate adds to diverse electrophiles to deliver the desired products while simultaneously regenerating the NHC catalyst.

**Scheme 14:**
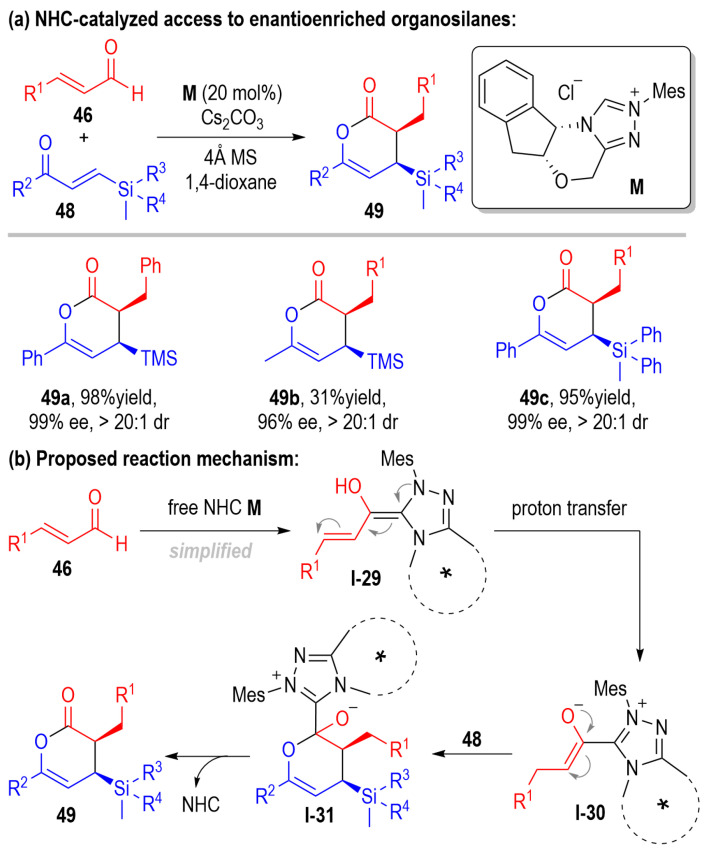
NHC-catalyzed asymmetric silylation of enals; the asterisk (*) denotes chirality.

In 2018, Huang, Fu, and co-workers developed a metal-free route to access optically enriched organosilanes **49** (31–98% yield) bearing two consecutive stereogenic centers. This method leveraged NHC organocatalysis to couple enals **46** with β-silyl enones **48** under mild reaction conditions, achieving moderate to excellent diastereo- and enantioselectivity across a broad substrate scope (Scheme 14a) [42]. The proposed mechanism is initiated by a nucleophilic attack of the free NHC catalyst **M** (20 mol%) on enal **46**, furnishing the key Breslow intermediate **I-29**. Intramolecular proton transfer of **I-29** then generates **I-30**, which undergoes an *endo*-Diels–Alder cycloaddition with β-silyl enone **48** to form the cyclized intermediate **I-31**. Subsequent elimination of the NHC catalyst affords the enantioenriched *cis*-organosilane **49** bearing two contiguous stereocenters, thereby completing the catalytic cycle (Scheme 14b).

**Scheme 15:**
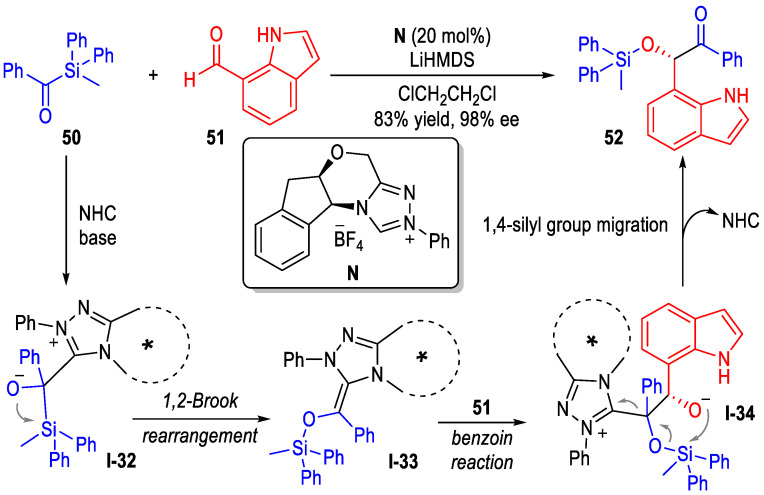
NHC-catalyzed asymmetric Brook-benzoin reaction; the asterisk (*) denotes chirality.

Expanding the scope of NHC-catalyzed umpolung beyond carbaldehydes, benzoylsilanes have recently been shown to be highly effective partners (Scheme 15) [43]. Employing NHC **N** (20 mol%) as the organocatalyst and LiHMDS as the base, benzoylsilane **50** reacts with indole-7-carbaldehyde **51** via a Brook-benzoin cascade to afford chiral silyl ether **52** in 83% yield and 98% ee value. Mechanistically, the NHC catalyst **N** preferentially activates benzoylsilane **50** over the aldehyde **51** under basic conditions, forming the adduct **I-32**. This adduct subsequently undergoes Brook rearrangement to yield the pivotal silyl-Breslow intermediate **I-33**, which then engages in a benzoin condensation with the indole aldehyde **51**. The resulting intermediate **I-34** finally delivers the product **52** through a 1,4-silyl migration, concurrently regenerating the NHC catalyst.

### 3.2. NHC-Catalyzed Synthesis of Silicon-Stereogenic Derivatives

Although several examples of NHC-catalyzed methodologies for synthesizing carbon-stereogenic silicon compounds have been reported, the construction of enantiomerically enriched silicon-stereogenic organosilicon molecules remains considerably more challenging.

**Scheme 16:**
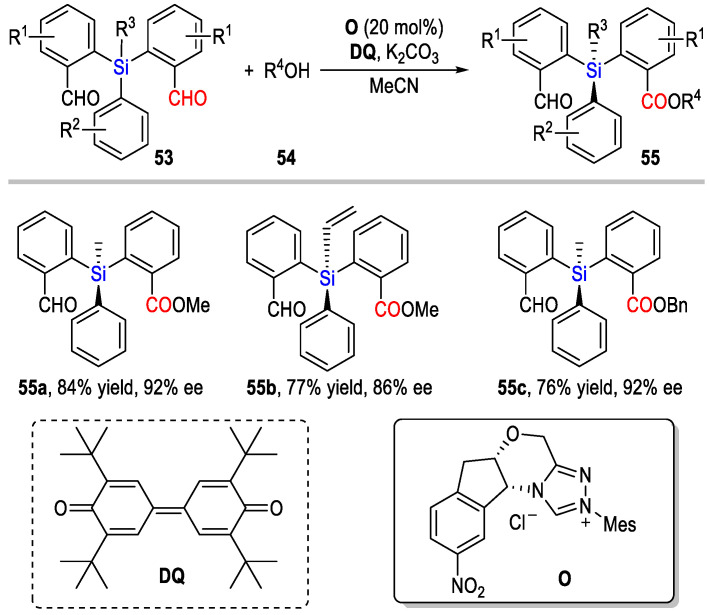
NHC-catalyzed desymmetrization reactions for the construction of stereogenic silane centers.

In 2022, Chi, Zheng, and co-workers disclosed an NHC-catalyzed oxidative strategy for the asymmetric synthesis of chiral tetrasubstituted silicon-stereogenic silanes **55** (Scheme 16) [44]. This approach employed a symmetric dialdehyde silane **53** as the substrate, which reacted with diverse alcohols **54** to afford the products in 76–84% yields with 86–97% ee values. A single-crystal diffraction study revealed that the substrate exists as a racemate of rapidly interconverting conformational enantiomers. This dynamic process is stabilized by a Si/O interaction, as confirmed by DFT calculations. The NHC catalyst **O** (20 mol%) selectively discriminated one conformer via a desymmetrization process to deliver the optically enriched monoester products in moderate to good yields with good to excellent optical purities. Notably, the chiral silanes obtained from this protocol could serve as versatile precursors for accessing a diverse range of other silicon-stereogenic functional molecules.

**Scheme 17:**
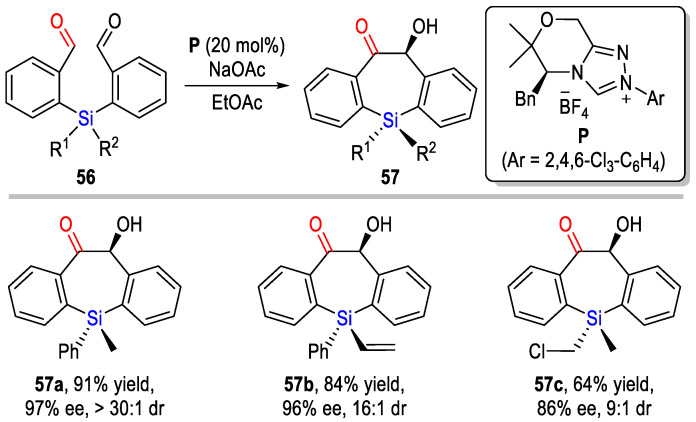
NHC-catalyzed intramolecular benzoin condensation for the construction of stereogenic silicon center.

Almost at the same time, Xu, Yang, Chen and co-workers reported an organocatalytic desymmetrization strategy for constructing optically active silicon-stereogenic silacycles **57** (Scheme 17) [45]. Employing the chiral NHC catalyst **P** (20 mol%), this method successfully discriminated between two identical formyl groups on the tetrasubstituted silanes **56**, enabling a stereoselective intramolecular benzoin reaction. This process afforded various enantioenriched dibenzo[*b*,*f*]silepin-10-ones **57** bearing both carbon and silicon stereocenters in 64–91% yields and optical purities. Furthermore, the catalytic protocol is readily scalable to the gram scale, and the obtained products could be employed as versatile precursors for further transformations.

## 4. Conclusions and Perspectives

NHC organocatalysis has revolutionized the synthesis of organosilicon derivatives, providing robust and versatile strategies for accessing these valuable compounds. Employing well-designed NHC precursors based on imidazolium, triazolium and thiazolium salts, this field has developed highly efficient methods for fundamental transformations like silylation, silylacylation, desymmetrization, and benzoin condensation via electron-pair or SET pathway. These advancements facilitate the direct construction of a broad spectrum of functionalized organosilicon molecules, with excellent control over both racemic and optically pure outcomes.

Despite significant progress in NHC-catalyzed organosilicon synthesis, limitations persist in the realms of catalyst design, substrate scope, and product utility. Firstly, the structural diversity of NHCs in this field remains limited. Breaking free from conventional scaffolds is essential to unlock novel reactivities and improve catalytic efficiency. Secondly, the scope of silicon reagents remains narrow, dominated by the activated species such as TMS-CN, a key challenge is the direct utilization of unactivated hydrosilanes. Furthermore, the product scope is largely restricted to silyl ethers, esters, ketones, and simple carbocycles. The strategic construction of silicon-stereogenic centers and the incorporation of silane motifs into complex architectures like functional polymers or bioconjugates remain significant and underexplored.

In summary, the application of NHC organocatalysis is highly efficient for the facile synthesis of organosilicon derivatives. Despite the significant success achieved in developing novel reactions and mechanistic understanding, continuous efforts are greatly desired regarding innovations in catalyst design, expansion of silicon partner scope, and the creation of structurally complex and functionally diverse organosilicon products.

## Data Availability

No new data were created or analyzed in this study. Data sharing is not applicable to this article.
